# Variations of *Escherichia coli* O157:H7 Survival in Purple Soils

**DOI:** 10.3390/ijerph14101246

**Published:** 2017-10-18

**Authors:** Taoxiang Zhang, Suping Hu, Wenhao Yang

**Affiliations:** 1 College of Forestry, Fujian Agriculture and Forestry University, Fuzhou 350002, China; xsnzheda2009@163.com (T.Z.); a199905@163.com (S.H.); 2 Fujian Provincial Key Laboratory of Soil Environmental Health and Regulation, College of Resources and Environment, Fujian Agriculture and Forestry University, Fuzhou 350002, China

**Keywords:** *Escherichia coli* O157:H7 (*E. coli* O157:H7), purple soils, Weibull model, soil nutrition, iron and aluminum oxide

## Abstract

*Escherichia coli* O157:H7 is a well-recognized cause of human illness. Survival of *Escherichia coli* O157:H7 in five purple soils from Sichuan Province was investigated. The dynamics of *E. coli* O157:H7 survival in purple soils were described by the Weibull model. Results showed that this model is suitable to fit survival curves of *E. coli* O157:H7 in purple soils, with the calculated *t_d_* value (survival time needed to reach the detection limit of 100 CFU·g^−1^) ranging from 2.99 days to 26.36 days. The longest survival time of *E. coli* O157:H7 was observed in neutral purple soils (24.49 days), followed by alkalescent purple soil (18.62 days) and acid purple soil (3.48 days). The redundancy analysis (RDA) revealed that *t_d_* values were significantly enhanced by soil nutrition (total organic carbon (OC), total nitrogen (TN), available potassium (AK) and the ratio of humic acid to fulvic acid (Ha/Fa)), but were significantly suppressed by iron and aluminum oxide.

## 1. Introduction

*Escherichia coli* O157:H7 is a food-borne pathogen that can cause hemorrhagic colitis and hemolytic uremic syndrome and has been detected with increasing frequency since 1982 [1]. Although most outbreaks of *E. coli* O157:H7 infections have been linked to foods of bovine origin, such as undercooked ground beef and dairy products [2], in recent decades, an increasing number of outbreaks caused by *Escherichia coli* O157:H7 have been associated with the consumption of fresh produce [3,4]. Produce can be contaminated at any point during the primary production through exposure to manure or contaminated irrigation water [5]. In 2006, there was a spinach contamination incident in the USA which was a multistate (26 states) outbreak of *Escherichia coli* O157:H7 that caused 199 illnesses and at least three deaths [6]. In another instance, an outbreak of *E. coli* O157:H7 infection among members of four families was associated with vegetables fertilized with cattle manure on the farm [7]. In America, the consumption of fresh produce contaminated by *E. coli* O157:H7 is responsible for a large portion of pathogen infections [8].

Cattle is considered to be the main environmental reservoir for *E. coli* O157:H7, with infected animals typically excreting between 10^2^ and 10^5^ CFU of *E. coli* O157:H7 g^−1^ faeces [9]. The land application of raw manure as a crop fertilizer or soil amendment potentially spreads pathogens to the agricultural environment [7,9,10]. This is of concern as the pathogen has been shown to persist for extensive periods of time in waste and waste-amended soil [9,11,12]. It has been testified that *E. coli* O157:H7 can be internalized into the root, leaf and stem of plant during sprouting and growth in soil contaminated by manure [3,5,10,11], which has created a serious public health risk. Soil has become a reservoir or vehicle of *E. coli* O157:H7 to the fresh vegetables, fruits and drinking water, hence, it is important to understand the persistence behavior of *E. coli* O157:H7 and its affecting factors in soils.

As a general observation, the population sizes of *E. coli* in soil and soil-related (manure) habitats have shown progressive declines in all habitats studied [3,4,7,13,14]. Ma et al. based on their research findings, stated that *E. coli* O157:H7 survived for about 35 days in soils from Salinas before reaching the detection limit (*t_d_*) [13]. Our previous findings showed that the average survival time of *E. coli* O157:H7 in soils from Jiangsu Province where the largest *E. coli* O157:H7 outbreak in China occurred were between 4.57–34.34 days [15]. The survival of *E. coli* O157:H7 in soil is affected by numerous soil properties and environment factors. A recent article on survival of *E. coli* O157:H7 in the outside environment (soil, manure and water) showed that availability of resources, pH, availability of water and temperature were the most important factors affecting survival [13,14,15]. The availability of resources such as carbon substrates is probably the main critical factor that affects the persistence of *E. coli* in open environments such as soil and water [16]. Soil organic carbon, total nitrogen, soluble organic carbon/nitrogen and clay which can provide the easily available energy sources for pathogen growth and decrease the competitive pressure between organisms, possibly allowing and enhancement of the persistence of *E. coli* O157:H7 in nutrient-rich soil environments [10,17]. 

In this study, the survival of *E. coli* O157:H7 in three kinds of purplish soils (alkalescent purple soil, neutral purple soil and acid purple soil) from Sichuan Province was investigated. Purplish soils are the mainly agricultural soil which accounts for 68.7% the total cultivated area of Sichuan Province [18]. The purple soil is of particular interest as it has a unique humid climate soil type, and its nutrient is heavily influenced by its parent material [19,20]. There have been no outbreaks of *E. coli* O157:H7 reported in Sichuan recently, but some sporadic cases occur in rural areas [21]. The presence of *E. coli* O157:H7 had been tested in external environments, such as excrements, sewages, foods and dairy cattle [21]. The sporadic detection of *E. coli* O157:H7 in environmental media implies that it is crucial to assess the potential risk of pathogen contamination in purple soil, and to develop control strategies to prevent the outbreak of disease in Sichuan. The objectives of this study were to gain insights into the *E. coli* O157:H7 survival dynamics in purple soil and identify the physical and chemical soil characteristics which are responsible for the difference in decline kinetics.

## 2. Materials and Methods

### 2.1. Soil Collection and Characterization

**Figure 1:**
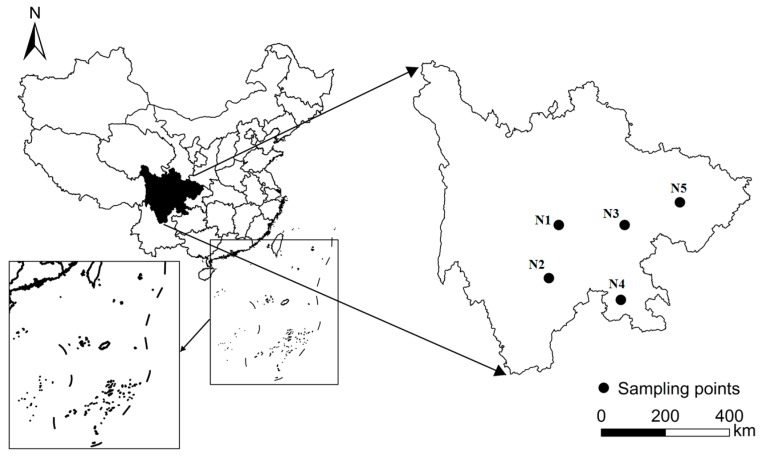
The map of sampling locations in Sichuan Province of China.

Soils were collected from five places throughout Sichuan Province, representing the three main kinds of purple soils in this province, including one sample of alkalescent purple soil (pH > 7.5), two of neutral purple soil (6.5 < pH < 7.5) and two of acid purple soil (pH < 6.5). The sites of each sample are shown in the map of sampling in Sichuan (Figure 1).

Fresh soils (0–20 cm) were stored at 4 °C in the refrigerator before conducting the study. Physical and chemical properties, including pH, organic carbon content (OC), total nitrogen (TN), total organic carbon (TOC), available potassium (AK), available phosphorus (AP), clay, silt and sand content, humus content, free iron and free aluminum were tested using air-dried soil by the standard methods described by the Agricultural Chemistry Committee of China [22], (Table 1).

### 2.2. Bacterial Strains

A rifampicin-resistant *E. coli* O157:H7 strain (EDL933) was used. This strain cannot produce the shiga-like toxin I or II (stx1 and stx2). In addition, there were no differences of survival between toxin-positive and toxin-negative *E. coli* O157:H7. Firstly, the frozen bacteria were activated in Luria-Bertani (LB) broth (Becton Dickinson, Franklin Lakes, NJ, USA) overnight at 37 °C, then 1 mL of bacterial suspension was further incubated in LB for 12 h (200 rpm) to achieve the early stationary phase of the bacteria. Cells were harvested by centrifugation, and then washed three times with phosphate buffer (10 mM, pH 7.2). The washed cells were suspended in sterile deionized water. Afterwards, the cell concentration of the washed cell suspension was determined by spread plate method on Sorbitol MacConkey Agar (SMAC). 

### 2.3. Survivals of Escherichia coli O157:H7 in Soils

The procedures of *Escherichia coli* O157:H7 inoculation, extraction and counting in soils were described previously by Zhang et al. [15]. Briefly, soil samples saved in a 4 °C refrigerator were kept at 25 ± 1 °C for one week to restore soil microbial activity before the incubation experiment. *E. coli* O157:H7 cells were inoculated into soil to a final concentration of 10^7^ CFU (colony forming units) per gram of dry soil (CFUg^−1^). The inoculated soil-bacteria mixtures were incubated at 25 °C throughout the incubation period. Three replicates of soil with and without inoculation of *E. coli* O157:H7 were conducted in each treatment. To ensure similar water availability in different soils, the water content of each individual samples was adjusted to 70% of the field moisture capacity, and maintained constantly by adding water to make up for the loss. At 0, 0.25, 1, 2, 3, 5, 7, 10, 15, 20, 25 and 30 days, 0.5 g (oven-dry basis) soils were sampled to extract the living bacteria and enumeration until no organisms could be tested. There were no detectable background *E. coli* O157:H7 populations in the soils prior to inoculation.

### 2.4. Survival Data Modeling

All data used to the model were average of triplicate measurements expressed as log_10_ CFU per gram of soil for each treatment-time combination. Survival of *E. coli* O157:H7 was modeled by fitting the experimental data to the Weibull survival function (Equation (1)) which has been well described [15]. This model is based on the assumption that the cells resistance to stress follows a Weibull distribution, and the survival curve is the cumulative form of this underlying distribution of individual inactivation kinetics:

log_10_(N_t_) = log_10_(N_0_) − (t/δ)^p^ (1)

### 2.5. Statistical Analysis

The data analysis was performed using Microsoft Excel 2007. The relationships between soil physical, chemical properties and *t_d_* (day) were determined by redundancy analysis (RDA direct), using CANOCO v.4.5 for Windows [23].

## 3. Results and Discussion

### 3.1. Model Performance

**Figure 2:**
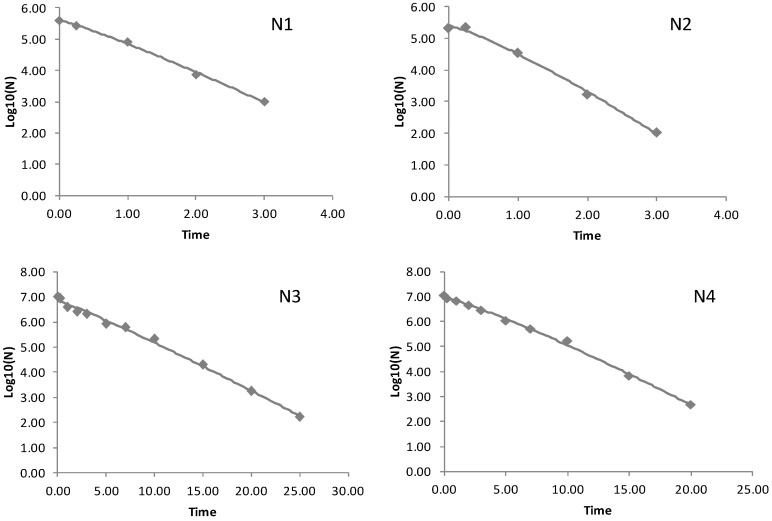

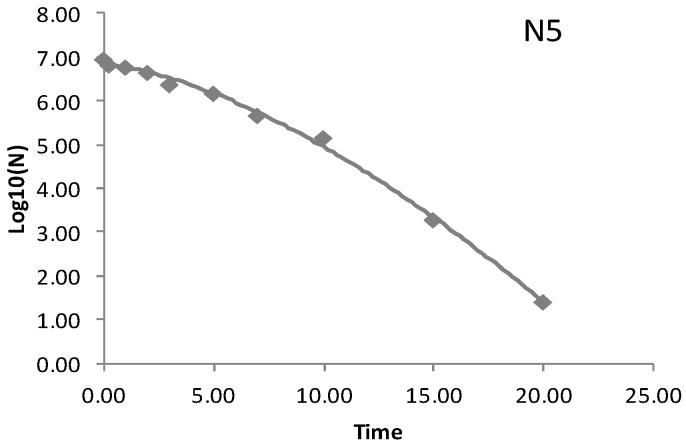
Weibull decline curve of survival of *E. coli* O157:H7 in the different soils N1–N5. Observed values (◆) and fitted Weibull decline curve (solid line).

**Figure 3:**
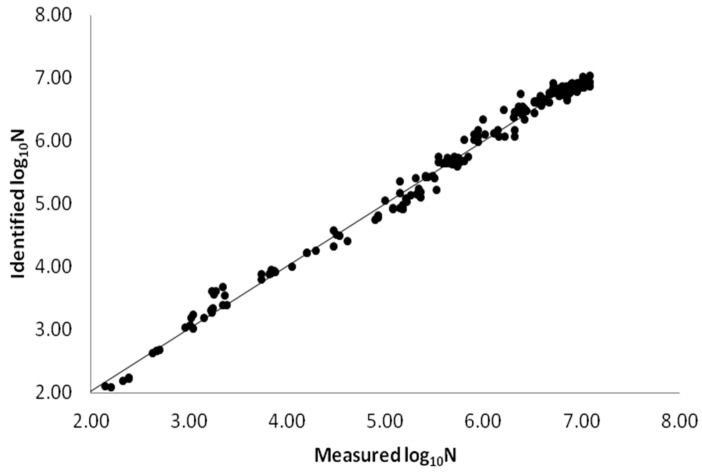
Plot of the correlation between observed and modelled values for log_10_N for survival in soils.

All of the survival curves showed a convex curvature (Figure 2), and the Weibull model for survival in five soils had a mean R^2^ of 0.98. Further analysis of the Weibull model parameters (log *N_t_*) indicated that the observed and modeled values of *E. coli* O157:H7 density over time were highly correlated (r = 0.96, *p* < 0.0001) (Figure 3), indicating that the Weibull model is suitable to fit survival curves of *E. coli* O157:H7 in purple soils. The model is sufficiently flexible to be responsible for different survival patterns (When *p* > 1, a convex curve is observed when *p* < 1, a concave curve is observed, and when *p* = 1, a linear curve is observed) and it has been previously used to model the decline of *E. coli* O157:H7 in manure-amended soil [10]. A concave curve (*p* < 1) would mean that the remaining cells have the ability to adapt to the applied stress resulting in the decreasing stress for the survival cell of pathogen. A linear survival curve (*p* = 1) means that the probability of dying does not depend on time, in other words, each cell is equally susceptible no matter how long the treatment lasts [24]. In the present study, the average value of *p* is larger than 1 (*p* = 1.20) which would mean that the cells become increasingly susceptible to stress, and the accumulated damage made the destruction rate increasing with time [24,25]. This phenomenon might be result of the encountered stress when cells exposed to other environments [25], and the limited nutrient supply or organic matter in soil cannot satisfy the metabolic requirement of *E. coli* O157:H7 [26]. Meanwhile, the competitive microbial community in soils might change over time, resulting in the probability of increasing stress for the surviving cells of *E. coli* O157:H7 [25].

### 3.2. Fate Behavior of Escherichia coli O157:H7 in Soils

The *E. coli* O157:H7 population dropped directly after inoculation by 0.2, 0.34 and 1.5 log CFU/g in neutral, alkalescent and acid purple soils, respectively (Figure 2). A similar phenomenon was found by Franz et al. who reported that *E. coli* O157:H7 populations in manure-amended soil declined immediately after inoculation by approximately 1.5 log CFU gdw^−1^ [27]. The principle is not clear until now, and we assumed that the instant decline effect may be induced by the adsorption of *E. coli* O157:H7 onto soil minerals. Studies have proved that the adsorption can be finished in short time [28], and the adsorption will gradually decrease with the rise of pH [29]. Meanwhile, the adsorption between *E. coli* O157:H7 and soil particles can lead to the loss in activity or viability of the bacteria [30], so *E. coli* O157:H7 populations dropped more in acid purple soil than in neutral and alkalescent purple soil.

**Figure 4:**
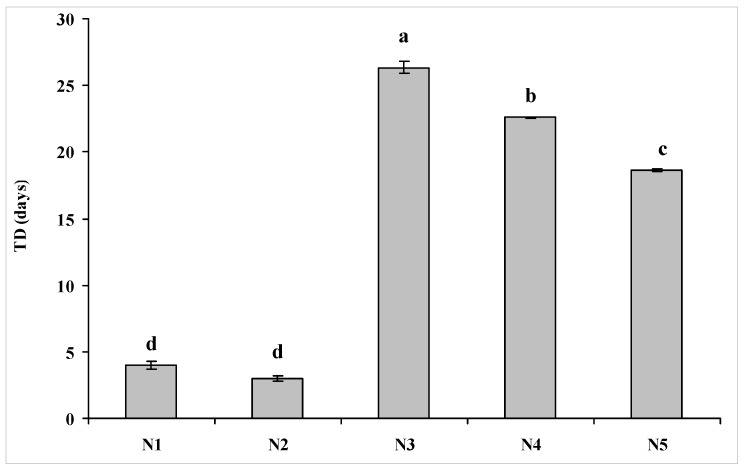
The survival time needed to reach the detection limit (*t_d_*, in days) for *E. coli* O157:H7 in the test soils. Bars are mean ± 1 standard deviation. Different letters on the error bars indicate significant differences at *p* < 0.05.

**Figure 5:**
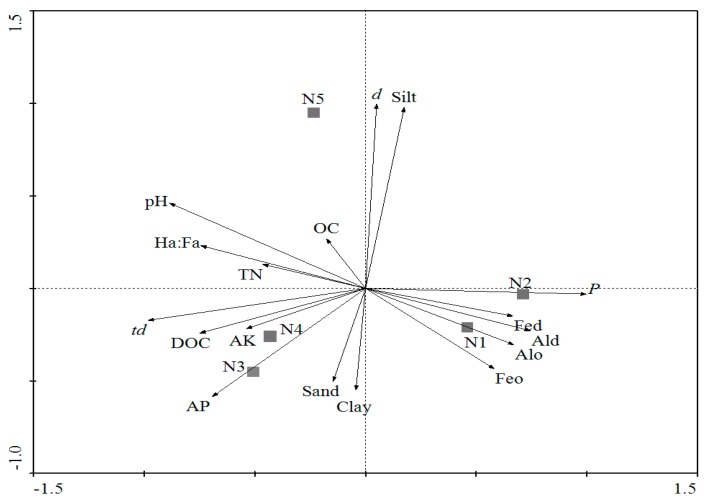
The redundancy analysis (RDA) of soil properties and the survival time (*t_d_*) of *E. coli* O157:H7 in the test soils.

*Escherichia coli* O157:H7 can survive for 24.50 days before reaching the detection limit in neutral purple soils, followed by alkalescent purple soil (18.62 days), and acid purple soils (3.64 days). A significant difference was observed among survival of *E. coli* O157:H7 (*t_d_*) in the different kinds of purple soils. *Escherichia coli* O157:H7 survived significant longer in neutral and alkalescent purple soil than in acid purple soil (Figure 4). RDA analysis (Figure 5) was performed to visualize survival profiles of *E. coli* O157:H7 in the soils, which revealed a huge difference in survival of *E. coli* O157:H7 among the three kinds of purple soil (Figure 4). This phenomenon is might be a result of the soil–forming progress of the different purple soils. Purple soil is developed from purple sand shale in subtropical and tropical climate conditions. Different soil parent rocks and hydrothermal conditions define the distinct pH and nutrient status of purple soil [19,20]. Among the three kinds of purple soil, neutral purple soil has higher soil fertility level and mineral nutrition, which provides greater capacity for supplying and keeping nutrients [31]. We surmised that the better fertilized conditions of neutral purple soils resulted in the observed longer survival time of pathogens than seen with alkalescent and acid purple soils.

### 3.3. RDA Analysis of Survival Data on Soil Properties

RDA analysis was conducted to determine the effects of soil properties on the survival of *E. coli* O157:H7. The results (Figure 5) showed that the survival of pathogen (*t_d_*) was positively related to soil pH, nutrient status including DOC, AK, TN and Ha/Fa, indicating that a higher level of pH and nutrient status might correspond to the longer survival time (*t_d_*) for *E. coli* O157:H7 in soils. This result had been verified by many studies [7,9,10,14,16]. *E. coli* O157:H7 is an intestinal pathogen, but neutral and alkaline environments, which not only provide more available nutrients for *E. coli* O157:H7 [9,10,11,16], but also reduce the toxicity of Al and Mn in soils [15,32], are more suitable for its survival. DOC, TN, and AK can contribute significantly to the cycling of soil nutrients which are substrates for microbial growth [10,14,15,17], so nutrients promote the growth of introduced pathogens and reduce the competitive pressure between the introduced pathogen and indigenous microbes, and finally impact the increasing persistence of *E. coli* O157:H7.

Ha/Fa represents the fertility and degree of humification of soil. Both humic acids and fulvic acid can provide nutrition and help form soil aggregates, which may benefit the survival of *E. coli* O157:H7. Meanwhile, long-term survival of *E. coli* O157:H7 might require low concentrations of heavy metals [33]. Humic acids, with larger molecular masses and various functional groups than fulvic acid, can undergo diverse mutual interactions with heavy metal in soil, and render the metals immobile to help in the heavy metal detoxification process [34]. It has been proved that humic acid can combine with free Cd, resulting in a reducing of ion content, while fulvic acid can dissolve the heavy metal Cd by combining with minerals, increasing the persistence of Cd heavy metal in the soil [35]. Hence, a larger portion of humic acids can favor the survival of *E. coli* O157:H7 by the adsorption and detoxification of heavy metals.

From this research, the survival of *E. coli* O157:H7 (*t_d_*) was negatively associated to iron and aluminum oxide (Fe_d_, Al_d_, Fe_o_ and Al_o_) in the purple soils (Figure 5). In acid soils, the content of iron and aluminum oxide is high, which is harmful to plant and microbes [36]. A recent study showed that iron and aluminum oxide may negatively influence the survival of *E. coli* O157:H7, and metal toxicity has been described as particularly important in the die-off of *E. coli* O157:H7 [37]. Iron and aluminum oxide are positively charged in soils, especially in acid soils, and iron and aluminum oxide can precipitate and coat clay surfaces at low pH by decreasing the critical coagulation concentration, clay dispersion, water uptake, and clay swelling [38]. Meanwhile, *E. coli* O157:H7 are negatively charged in most natural soils. Previous studies proved that the coating of iron and aluminum oxide help the adhesion of bacteria to soil minerals [39,40], therefore, a high content of iron and aluminum oxide not only poisons *E. coli* O157:H7, but also promotes *E. coli* O157:H7 adhesion to soil minerals, thus weakening the survivability of *E. coli* O157:H7 in soil.

## 4. Conclusions

This research showed how *E. coli* O157:H7 survive in the purplish soils of Sichuan Province of China. A significantly longer *E. coli* O157:H7 survival was observed in neutral and alkalescent purple soils than in acid purple soils. *E. coli* O157:H7 can survive in purple soil for up to 24.50 days, which means the accumulation time of *E. coli* O157:H7 in purple soils through fertilization and wastewater irrigation is longer, so there is a risk of infection with *E. coli* O157:H7 if people or animals eat contaminated vegetables and fruits in the soil or exposed to contaminated soil. Furthermore, the soil with *E. coli* O157:H7 not only contaminates surface waters though rain-wash, but also the migratory *E. coli* O157:H7 bacteria can migrate from the soil to groundwater and result in the contamination of underground drinking water sources. The common tillage and the first rainfall after fertilization can promote the migration of pathogenic bacteria and transfer within hours a large number of pathogenic bacteria into the groundwater, where it can remain active for several months. In this experiment, *E. coli* O157:H7 could survive more than 15 days in neutral and alkalescent purple soils. Hence there is a risk of contamination of surface and groundwater water, and the risk of biological pollution should be taken seriously.

From this research, we found that the survival of *E. coli* O157:H7 in these soils was controlled by multiple factors. Soil pH, nutrient and iron and aluminum oxide were the main factors. The survival of *E. coli* O157:H7 were significantly enhanced by soil pH and nutrition (DOC, TN, AK and Ha/Fa), but was significantly suppressed by iron and aluminum oxide. Therefore, the survival of *E. coli* O157:H7 in soils with high pH and nutrition will be much longer than in other soils, and the risk of biological pollution should be taken seriously.

## Figures and Tables

**Table 1:** Physical and chemical properties of the soils used in this study.

Soil Code	pH	DOC	AK	AP	OC g kg^−1^	TN	Clay	Silt	Sand	Ha/Fa	Fe_d_	Al_d_	Fe_o_	Al_o_
		mg·kg^−1^					%				g·kg^−1^			
N1	4.56	9.52	112.54	0.26	8.6	0.89	38.30	37.27	24.43	0.16	44.14	11.34	4.38	4.92
N2	3.89	28.11	48.76	1.69	12.8	0.88	25.53	39.00	35.47	0.36	23.34	5.43	1.70	2.47
N3	6.63	62.79	349.89	15.97	16.3	1.77	29.93	33.03	37.04	0.66	19.18	2.54	1.66	1.48
N4	6.64	38.94	51.47	7.62	6.9	0.65	35.07	33.83	31.10	0.42	8.00	1.10	0.55	1.80
N5	7.84	32.65	143.94	0.68	14.4	1.38	25.82	48.08	26.10	0.58	17.10	1.97	0.33	1.31

Total soil organic carbon (OC); total soil nitrogen (TN); available potassium (AK); available phosphorus (AP); dissolved organic carbon (DOC); the ratio of humic acid to fulvic acid (Ha/Fa); free Fe_2_O_3_(Fe_d_); free Al_2_O_3_(Al_d_); amorphous Fe_2_O_3_(Fe_o_); amorphous Al_2_O_3_(A_o_).

## References

[1] Keene W.E., McAnulty J.M., Hoesly F.C., Williams L.P., Hedberg K., Oxman G.L., Barrett T.J., Pfaller M.A., Fleming D.W. (1994). A swimming-associated outbreak of hemorrhagic colitis caused by *Escherichia coli* O157:H7 and Shigella sonnei. N. Engl. J. Med..

[2] Feng P. (1995). *Escherichia coli* serotype O157:H7: Novel vehicles of infection and emergence of phenotypic variants. Emerg. Infect. Dis..

[3] Oliveira M., Viñas I., Usall J., Anguera M., Abadias M. (2012). Presence and survival of *Escherichia coli* O157:H7 on lettuce leaves and in soil treated with contaminated compost and irrigation water. Int. J. Food. Microbiol..

[4] Semenov A.V., van Overbeek L., van Bruggen A.H. (2009). Percolation and survival of *Escherichia coli* O157:H7 and Salmonella enterica serovar Typhimurium in soil amended with contaminated dairy manure or slurry. Appl. Environ. Microb..

[5] Solomon E.B., Yaron S., Matthews K.R. (2002). Transmission of *Escherichia coli* O157:H7 from contaminated manure and irrigation water to lettuce plant tissue and its subsequent internalization. Appl. Environ. Microb..

[6] Eppinger M., Mammel M.K., Leclerc J.E., Ravel J., Cebula T.A. (2011). Genomic anatomy of *Escherichia coli* O157:H7 outbreaks. Proc. Natl. Acad. Sci. USA.

[7] Jiang X., Morgan J., Doyle M.P. (2002). Fate of *Escherichia coli* O157:H7 in manure-amended soil. Appl. Environ. Microb..

[8] Scallan E., Hoekstra R.M., Angulo F.J., Tauxe R.V., Widdowson M.-A., Roy S.L., Jones J.L., Griffin P.M. (2011). Foodborne illness acquired in the United States-major pathogens. Emerg. Infect. Dis..

[9] Williams A., Avery L., Killham K., Jones D. (2007). Survival of *Escherichia coli* O157:H7 in the rhizosphere of maize grown in waste-amended soil. J. Appl. Microbiol..

[10] Franz E., Semenov AV., Termorshuizen A.J., De Vos O., Bokhorst J.G., Van Bruggen A.H. (2008). Manure-amended soil characteristics affecting the survival of *E. coli* O157:H7 in 36 Dutch soils. Environ. Microbiol..

[11] Ongeng D., Vasquez G., Muyanja C., Ryckeboer J., Geeraerd A., Springael D. (2011). Transfer and internalisation of *Escherichia coli* O157:H7 and Salmonella enterica serovar Typhimurium in cabbage cultivated on contaminated manure-amended soil under tropical field conditions in Sub-Saharan Africa. Int. J. Food Microbiol..

[12] Semenov A.V., van Overbeek L., Termorshuizen A.J., van Bruggen A.H. (2011). Influence of aerobic and anaerobic conditions on survival of *Escherichia coli* O157:H7 and Salmonella enterica serovar Typhimurium in Luria-Bertani broth, farm-yard manure and slurry. J. Environ. Manag..

[13] Ma J., Ibekwe A.M., Yang C.H., Crowley D.E. (2013). Influence of bacterial communities based on 454-pyrosequencing on the survival of *Escherichia coli* O157:H7 in soils. FEMS Microbiol. Ecol..

[14] Ongeng D., Muyanja C., Geeraerd A., Springael D., Ryckeboer J. (2011). Survival of *Escherichia coli* O157:H7 and Salmonella enterica serovar Typhimurium in manure and manure-amended soil under tropical climatic conditions in Sub-Saharan Africa. J. Appl. Microbiol..

[15] Zhang T., Wang H., Wu L., Lou J., Wu J., Brookes P.C., Xu J. (2013). Survival of *Escherichia coli* O157:H7 in soils from Jiangsu Province, China. PLoS ONE.

[16] Van Elsas J.D., Semenov A.V., Costa R., Trevors J.T. (2010). Survival of *Escherichia coli* in the environment: Fundamental and public health aspects. ISME J..

[17] Ma J., Ibekwe A.M., Crowley D.E., Yang C.-H. (2012). Persistence of *Escherichia coli* O157:H7 in major leafy green producing soils. Environ. Sci. Technol..

[18] Tang S., Sun D., Luo Y., Zhou D., He R., Mao J., Luo Y. (1984). The fertility of purple soil in relation to the characteristics of parent material in Sichuan Basin. Acta Pedol. Sin..

[19] Wang H., Shi X., Yu D., Weindorf D.C., Huang B., Sun W., Ritsema C.J., Milne E. (2009). Factors determining soil nutrient distribution in a small-scaled watershed in the purple soil region of Sichuan Province, China. Soil Till. Res..

[20] Zhu B., Wang T., You X., Gao M. (2008). Nutrient release from weathering of purplish rocks in the Sichuan Basin, China. Pedosphere.

[21] Zhang B., Liu H., Zhang C., Luan R., Tu G. (2005). Molecular epidemiolog y, antimicrobial and disinfectant resistance of *Escherichia coli* O157:H7 in Sichuan. J. Hygiene Res..

[22] Agricultural Chemistry Committee of China (1983). Conventional Methods of Soil and Agricultural Chemistry Analysis.

[23] Ter Braak C.J., Prentice I.C. (1988). A theory of gradient analysis. Adv. Ecol. Res..

[24] Peleg M. (2003). Microbial survival curves: Interpretation, mathematical modeling, and utilization. Comments Theor. Biol..

[25] Van Boekel M.A. (2002). On the use of the Weibull model to describe thermal inactivation of microbial vegetative cells. Int. J. Food. Microbiol..

[26] Crane B.S., Moore J. (1986). Modeling enteric bacterial die-off: A review. Water Air Soil Poll..

[27] Franz E., van Diepeningen A.D., de Vos O.J., van Bruggen A.H. (2005). Effects of cattle feeding regimen and soil management type on the fate of *Escherichia coli* O157:H7 and Salmonella enterica serovar Typhimurium in manure, manure-amended soil, and lettuce. Appl. Environ. Microb..

[28] Yee N., Fein J.B., Daughney C.J. (2000). Experimental study of the pH, ionic strength, and reversibility behavior of bacteria–mineral adsorption. Geochim. Cosmochim. Acta.

[29] Wu H., Jiang D., Cai P., Rong X., Dai K., Liang W., Huang Q. (2012). Adsorption of Pseudomonas putida on soil particle size fractions: Effects of solution chemistry and organic matter. J. Soil Sediments.

[30] Cai P., Huang Q., Walker S.L. (2013). Deposition and Survival of *Escherichia coli* O157:H7 on Clay Minerals in a Parallel Plate Flow System. Environ. Sci. Technol..

[31] Wang H., Xing X., Xu Z. (2007). Relationship between pH value and available nutrients of purple soil in panxi tobacco-growing areas. Soil Fertil. Sci. Chin..

[32] Aciego Pietri J.C., Brookes P.C. (2008). Relationships between soil pH and microbial properties in a UK arable soil. Soil Biol. Biochem..

[33] Artz R.R., Killham K. (2002). Survival of *Escherichia coli* O157:H7 in private drinking water wells: Influences of protozoan grazing and elevated copper concentrations. FEMS Microbiol. Lett..

[34] Pandey A.K., Pandey S.D., Misra V. (2000). Stability constants of metal–humic acid complexes and its role in environmental detoxification. Ecotoxicol. Environ. Saf..

[35] Dalang F., Buffle J., Haerdi W. (1984). Study of the influence of fulvic substances on the adsorption of copper (II) ions at the kaolinite surface. Environ. Sci. Technol..

[36] Robert M., Huang P., Berthelin J., Bollag J., McGill W., Page A. (1995). Aluminum toxicity: A major stress for microbes in the environment. Environmental Impact of Soil Component Interactions: Volume 2: Metals, other Inorganics, and Microbial Activities.

[37] Avery L., Williams A., Killham K., Jones D. (2008). Survival of *Escherichia coli* O157:H7 in waters from lakes, rivers, puddles and animal-drinking troughs. Sci. Total Environ..

[38] Goldberg S. (1989). Interaction of aluminum and iron oxides and clay minerals and their effect on soil physical properties: A review. Commun. Soil Sci. Plan..

[39] Chen G., Zhu H. (2005). Bacterial adhesion to silica sand as related to Gibbs energy variations. Colloid. Surf. B.

[40] Lee C.G., Park S.J., Han Y.U., Park J.A., Kim S.B. (2010). Bacterial attachment and detachment in aluminum-coated quartz sand in response to ionic strength change. Water Environ. Res..

